# *Plasmodium falciparum* glutamate dehydrogenase is genetically conserved across eight malaria endemic states of India: Exploring new avenues of malaria elimination

**DOI:** 10.1371/journal.pone.0218210

**Published:** 2019-06-14

**Authors:** Amreen Ahmad, Anil Kumar Verma, Sri Krishna, Anjana Sharma, Neeru Singh, Praveen Kumar Bharti

**Affiliations:** 1 ICMR-National Institute of Research in Tribal Health (NIRTH), Garha, Jabalpur, India; 2 Department of P. G. Studies and Research in Biological Science, Rani Durgavati University, Pachpedi, Jabalpur, Madhya Pradesh, India; Instituto Rene Rachou, BRAZIL

## Abstract

Accurate and timely diagnosis is very critical for management, control and elimination of the malaria. Malaria rapid diagnostic tests (RDTs) have improved the diagnosis and management of malaria in remote areas, community and places where microscopy is not available for diagnosis. According to WHO report 2018, *Plasmodium falciparum* malaria constitutes more than 50% of malaria cases in India. Most of the RDTs used for diagnosis of falciparum malaria today employ HRP2 as a target antigen. However, low density parasitemia and deletion of hrp-2 gene in *P*. *falciparum* leads to false negative results and necessitates the development of alternative/ new or improved RDT for malaria diagnosis. We have analysed the genetic diversity and homology modelling of *Pfgdh* (glutamate dehydrogenase), *ldh* (lactate dehydrogenase) and *aldolase* genes in *P*. *falciparum* isolates from the eight endemic states of India to assess their potential as antigen for RDT development. We observed negligible sequence diversity in *Pfgdh* in comparison to the low level of diversity in *ldh* and *aldolase* gene. No structural or functional changes were observed in modelling studies and all three genes were under negative purifying selection pressure. The highly conserved nature of *pfgdh* gene suggests that GDH could be a potential target molecule for Pan/Pf diagnostic test for malaria.

## Introduction

Malaria imposes heavy health and socioeconomic burden on tropical and sub-tropical regions of the world with an estimated 219 million cases and 435 000 deaths in 2017 [1]. India contributed about 85% of total malaria burden in the South East Asia Region [1]. Majority of malaria cases and deaths in India are reported from rural areas of the state with dominant tribal population. These include Odisha, Chhattisgarh, Jharkhand, Madhya Pradesh, Maharashtra, West Bengal, Karnataka, Assam Gujarat and North- Eastern (NE) states [2]. In these states, malaria endemic districts are located in hilly, densely forested, remote, inaccessible areas and are often marred by inadequate health care facilities. Lackadaisical health care seeking behaviour and socio-economic backwardness add further salt to injury [3]. The WHO has set the goal of malaria elimination by 2030 [1]. However, this aim can only be achieved when all the indigenous cases of malaria are diagnosed correctly and treated properly through the available diagnostic tools like microscopy, Rapid Diagnostic Test (RDT) and Polymerase Chain Reaction (PCR). Microscopy is considered ideal for malaria diagnosis but it has several limitations including requirement of skilled microscopist for interpretation of results and poor sensitivity in detecting low-level parasitaemia [4]. With RDT, it is possible to diagnose malaria instantly in the field, rural and poor health care settings where microscopy is not available and clinicians have to make quick decision for case management. The ease of handling and prompt diagnosis has made malaria RDTs more popular. Since, its introduction, the sales of malaria RDTs has grown tremendously with distribution of 1.92 billion RDT globally during 2010–2017. About 276 million RDT were sold in 2017 alone in the world. Due to high prevalence of malaria, the WHO Africa region was on the biggest consumer of RDT (223 million) in 2017 [1]. The sales and use of RDT has increased from 10.6 million to 15.2 million with the total use of 118.1 million RDT units for malaria diagnosis during 2010–2017 in India, [1, 5].

*Pf*HRP2/3 based RDT are commonly used for diagnosis of falciparum malaria. *Pf*HRP3 is analogue of *Pf*HRP2 which share common antigenic epitope. At present out of 44 WHO recommended RDT, 41 RDTs target histidine-rich protein 2 (*Pf*HRP2) either alone or in combination with *pfldh* for diagnosis of *P*. *falciparum* or pan *Plasmodium* genera [6]. Moreover, a number of studies from various parts of the world including India have reported the complete or partial deletion of *Pfhrp2/3* gene [7–9]. The deletion of *Pfhrp2/3* gene has rendered HRP2 based RDTs ineffective for diagnosis of such strains of *P*. *falciparum* and correlates with the poor performance of HRP2-based RDTs [10].

According to WHO report of Malaria RDT product testing few RDTs use Pf-pLDH for diagnosis of *P*. *falciparum* [6]. The sensitivity and performance of pLDH based pan malaria RDT and/or *P*. *vivax* pLDH RDT was found to be satisfactory. Very few RDTs (ParaHIT) use pan-Plasmodium aldolase in combination with pfHRP-2 for diagnosis of malaria. The sensitivity of such RDTs were found to be poor because of relatively low expression of aldolase by the malaria parasite [11,12].

Concerns about the stability and performance of RDT (in terms of sensitivity and specificity) and antigen genetic diversity call for exploration and development of alternative or improved RDTs for malaria diagnosis [13–15]. Glutamate dehydrogenase (GDH) is considered a potential biomarker for *P*. *falciparum* diagnosis with high level of immunogenicity [16,17]. Glutamate dehydrogenase enzyme is a ubiquitous enzyme which plays important role in glutamate catabolism and ammonium assimilation [17,18]. It catalyses NAD(P)+ linked oxidative deamination reaction of L-glutamate to 2-oxoglutarate and ammonia. *P*. *falciparum* contains three genes encoding potential parasite glutamate dehydrogenase (pGDH) proteins; two genes are found on chromosome 14 (PF14_0164 and PF14_0286, encoding pGDH1 and pGDH2, respectively) and one on chromosome 8 (PF08_0132, encoding pGDH3). *P*. *falciparum* glutamate dehydrogenase (pfgdh1) is the main producer of NAD(P)+ in erythrocytes and is essential for maintenance of redox balance of the cell [19]. Moreover, PfGDH is exclusively expressed by malaria parasite in erythrocytic stages and remain localised in cytosol [20] The concentration of GDH directly correlates with parasite load [17,19, 21]. Therefore, it can be used as marker of *P*. *falciparum*. To our knowledge very little or no data is available on genetic polymorphism of *pfaldolase*, *pfldh* and *pfgdh* gene in Indian isolates [22]. Therefore, the present research is intended on analysis of the sequence variation in Glutamate dehydrogenase *(pfgdh)* gene, a potential diagnostic target for diagnosis of *P*. *falciparum* and its comparison with lactate dehydrogenase and aldolase genes.

## Material and methods

### Sample collection and study site

**Fig 1:**
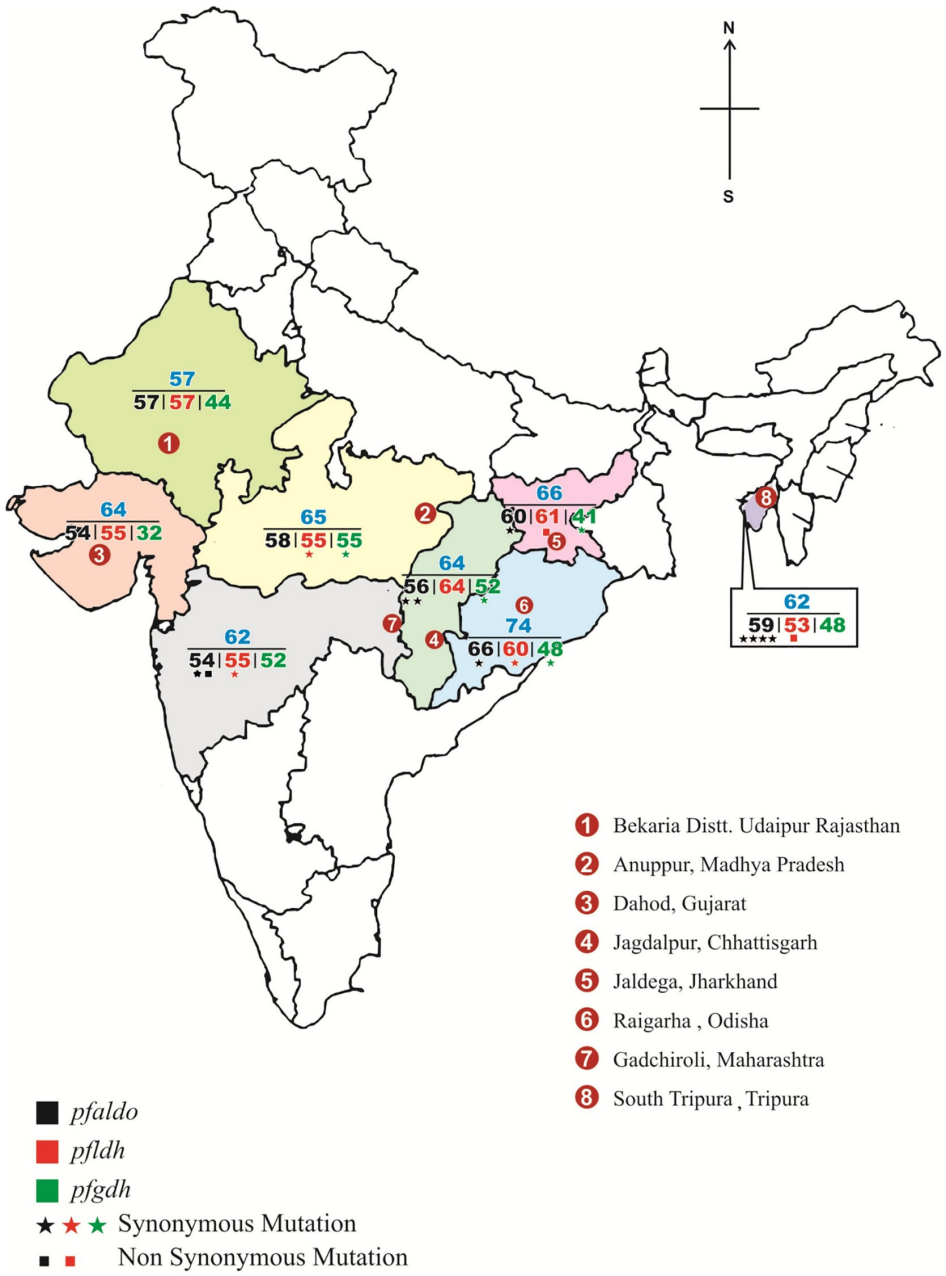
Map of India showing sampling site and number of samples used in the study. The sampling of blood samples was done at selected site from the endemic district of respective states as indicated in map.

The patient details and demographic information is already given in Bharti *et al* [9]. Blood samples collected in the study to explore deletion of *pfhrp2* and *pfhrp3* gene in eight endemic states of India were used to determine the genetic diversity of *pfgdh*, *pfldh* and *pfaldolase* genes (Fig 1). Ethical clearance was obtained from the Institutional Ethics Committee of National Institute of Research in Tribal Health (NIRTH), Jabalpur. Before collecting the samples, written informed consent was obtained from the patients or from the parents/guardian in case of children as per the guidelines of ICMR. Consent form was also provided and explained to the patients and parents/Guardian in case of children in local language.

### Genomic DNA isolation

Parasite DNA was extracted from the whole blood samples of patients using QIAamp Blood kit as per manufacturer protocol and purified DNA was used as a template in PCR.

### Molecular diagnosis by Polymerase Chain Reaction

Species-specific nested PCRs were performed in earlier study to confirm *Plasmodium falciparum* mono-infection [23].

#### PCR amplification of *pfgdh*, *pfldh* and *pfaldolase* gene

**Fig 2:**
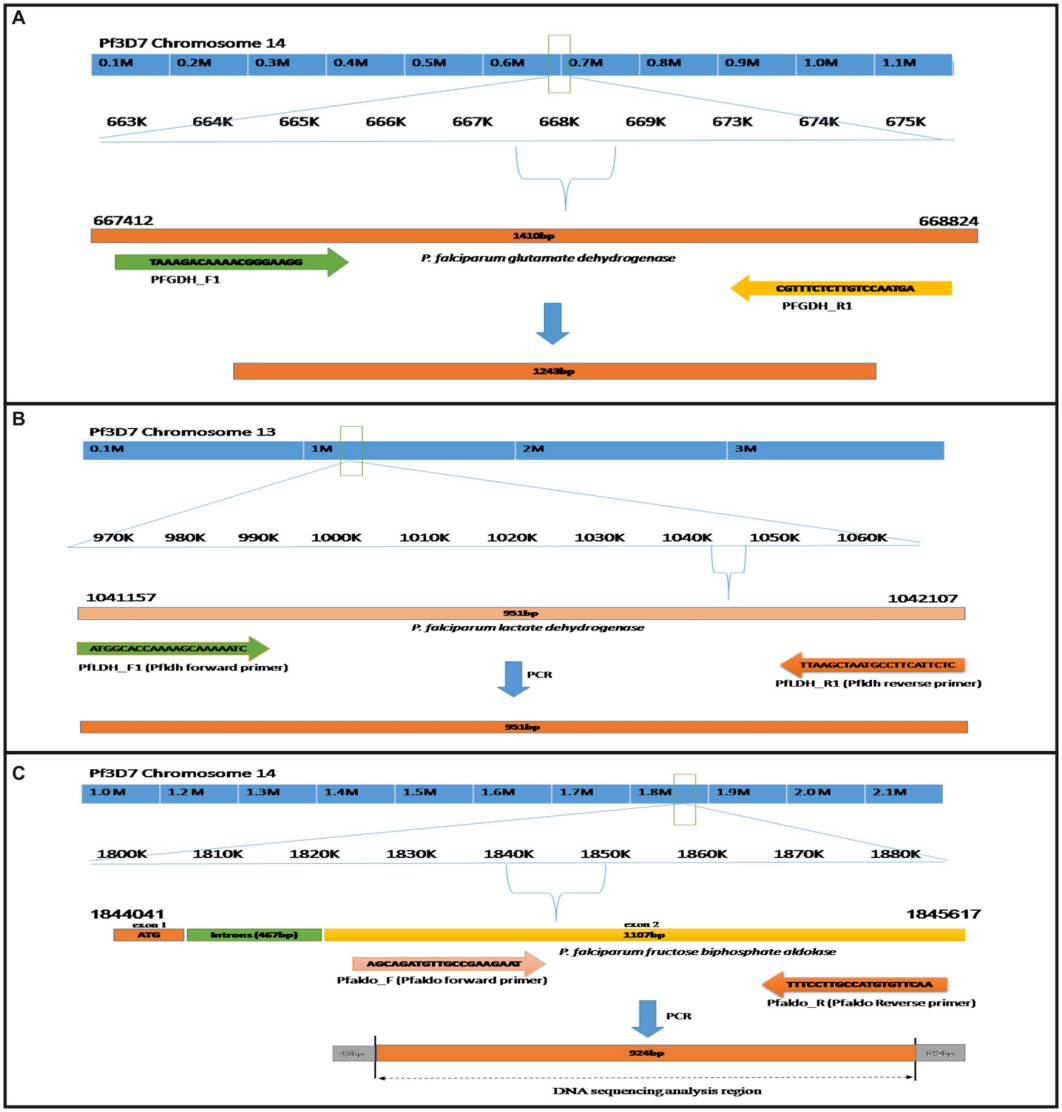
Schematic representation of primer binding site and location of (A) *P*. *falciparum* glutamate dehydrogenase (B) *P*. *falciparum* lactate dehydrogenase (C) *P*. *falciparum* aldolase gene in the chromosome.

Positive samples for *P*. *falciparum* were selected for the PCR amplification of *pfgdh*, *pfldh* and *pfaldolase* gene using primers and PCR conditions described in Table 1. The primer were designed to amplify *pfgdh* gene (PfGDH F1 and PfGDH R1), *pfldh* gene (PfLDH F1 and PfLDH R1) and exon 2 of the *Pf aldolase* gene (PfALDO F1 and PfALDO R1) as described in Fig 2, PCR reaction was carried out in a final reaction volume of 25μl reaction mixture containing 5μl of DNA template, 10X buffer, 0.4μM of each forward and reverse primers, 2mM MgCl_2_, 0.2mM each dNTPs and 0.2 units of Taq Polymerase (Invitrogen, life technologies). PCR products of all genes were resolved on a 1.2% agarose gel and image was captured using GelDoc-It^2^ imager. The 1kb DNA ladder (invitrogen) was used to determine the size of PCR products.

**Table 1:** Details of primer sequences, amplicon size and PCR conditions.

Gene	Primer Sequence (5’➔ 3’); Forward (F) and Reverse (R)	PCR product length	PCR conditions			No. of cycles
			Denaturation	Annealing	Extension	
***pfgdh***	PFGDH_F1- TAAAGACAAAACGGGAAGG	1243bp	95°C 30 sec	57°C 30 sec	72°C 30 sec	30
	PFGDH_R1- CGTTTCTCTTGTCCAATGA					
***pfldh***	PFLDH _F1- ATGGCACCAAAAGCAAAAATC	951bp	95°C 30 sec	58°C 30 sec	72°C 30 sec	30
	PFLDH_R1- TTAAGCTAATGCCTTCATTCTC					
***pfaldolase***	PFALDO_F1- AGCAGATGTTGCCGAAGAAT	924bp	95°C 30 sec	57°C 40 sec	72°C 30 sec	45
	PFALDO_R1- TTTCCTTGCCATGTGTTCAA					

### DNA sequencing and analysis

Samples that showed the positive amplification for *pfgdh*, *pfldh* and *Pfaldolase* genes were sequenced from both directions by using forward and reverse primers of respective genes. PCR products were purified by using spin columns (Real Biotech Corporation, Taiwan). The DNA sequencing of purified DNA was done using BigDye Terminator v3.1 Cycle Sequencing Kit on Applied Biosystems 3730 XL DNA analyser. Sequences were analysed using software v5.2 (Applied Biosystems) and were aligned using Bio Edit Sequence Alignment editor version 7.2.6.1 with the reference sequence of *pfgdh*, *pfldh* and *pfaldolase* gene available in the database. Nucleotide sequences of poor quality were not included in the final analysis. Nucleotide sequences were further translated to amino acid using ExPASy translate tool (https://web.expasy.org/translate/). Amino acid sequences were aligned with the reference sequence to look for significant and non-significant structural/functional changes in protein.

### Structural comparison

The model of protein structure having polymorphic amino acid sequences were generated by template based modelling in SWISS-MODEL [24] and the model was aligned with the reference crystal structure available in protein data bank (PDB) using PyMOL molecular graphic system (v1.7.4.5) to asses any structural changes in 3D structure of protein [25]. Model quality was estimated using QMEAN score (Qualitative Model Energy Analysis) and the rmsd value (root mean square deviation) [26, 27].

### Statistical analysis

Population genetic parameters were measured to quantify selection pressure using different methods in MEGA7 software [28]. Ratio of substitution rate at non synonymous and synonymous sites (dN/dS) was quantified using Nei and Gojobori (jukes—Contor) method [29, 30]. Average number of substitution (π) per site, hetrozygosity per site (θ) per site and Tajima test statistic (D) were estimated [31,32]. The number of transitional and transversional substitutions per site from over all sequence pairs were also calculated using the Tamura-Nei model [33].

## Results

A total of 514 microscopy and PCR positive samples (which were available in sufficient quantity) for *P*. *falciparum* were selected for present study from a previous PfHRP-2 study [9]. The sizes of *pfgdh*, *pfldh* and *Pfaldolase* amplified gene products were 1243bp, 924bp and 921bp respectively.

### Sequence analysis of *gdh* gene from *P*. *falciparum* Indian isolates

A total of 360 successfully sequenced samples yielded good quality sequences. These sequences were compared with the reference sequence (PF3D7_1416500) available in the NCBI database. Only two nucleotide substitutions were observed in five samples at position C 546 G and T 672 C (Table 2). None of these nucleotide substitutions led to change in amino acid. Amino acid sequences showed 100% identity with *P*. *falciparum* 3D7 (XP_001348337.1). Additional information about homology analysis with other similar sequences is given in S1 Table.

**Table 2:** Single nucleotide polymorphism and amino acid changes in *pfgdh*, *pfaldolase* and *pfldh* gene found in the study.

S. No.	Gene	SNP (by position and change)	Amino acid change (by position and change)	No. of sample with SNPs / total no. of samples
1.	***P*. *falciparum* glutamate dehydrogenase**	546 (ACC to ACG)	Synonymous	3/360
		672 (TCT to TCC)	Synonymous	2/360
**2**.	***P*. *falciparum* lactate dehydrogenase**	123 (CCA to CCG)	Synonymous	1/450
		159 (GTT to GTC)	Synonymous	1/450
		765 (AAA to AAG)	Synonymous	1/450
		814 (GAT to AAT)	272 (D to N)	2/450
**3**.	***P*. *falciparum* aldolase**	156 (AAC to AAT)	Synonymous	1/464
		234 (TTC to TTT)	Synonymous	1/464
		312 (CAC to CAG)	104 (H TO Q)	1/464
		444 (GCA to GCT)	Synonymous	1/464
		630 (GCA to GCG)	Synonymous	2/464
		729 (GCT to GCA)	Synonymous	1/464
		753 (ACC to ACT)	Synonymous	3/464
		819 (CCA to CCC)	Synonymous	1/464

D, Aspartate; H, Histidine; N, Aspargine; Q, Glutamine.

### Sequence and structure analysis of *ldh* gene from *P*. *falciparum* Indian isolates

**Fig 3:**
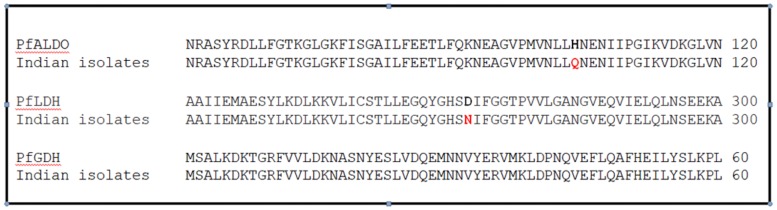
Multiple amino acid sequence alignment of *Paldolase* (XP_001348599.1), *Pfldh* (XP_001349989.1) and *Pfgdh* (XP_001348337.1) with test sequences using Clustal omega 1.2.4 multiple sequence alignment tool.

**Fig 4:**
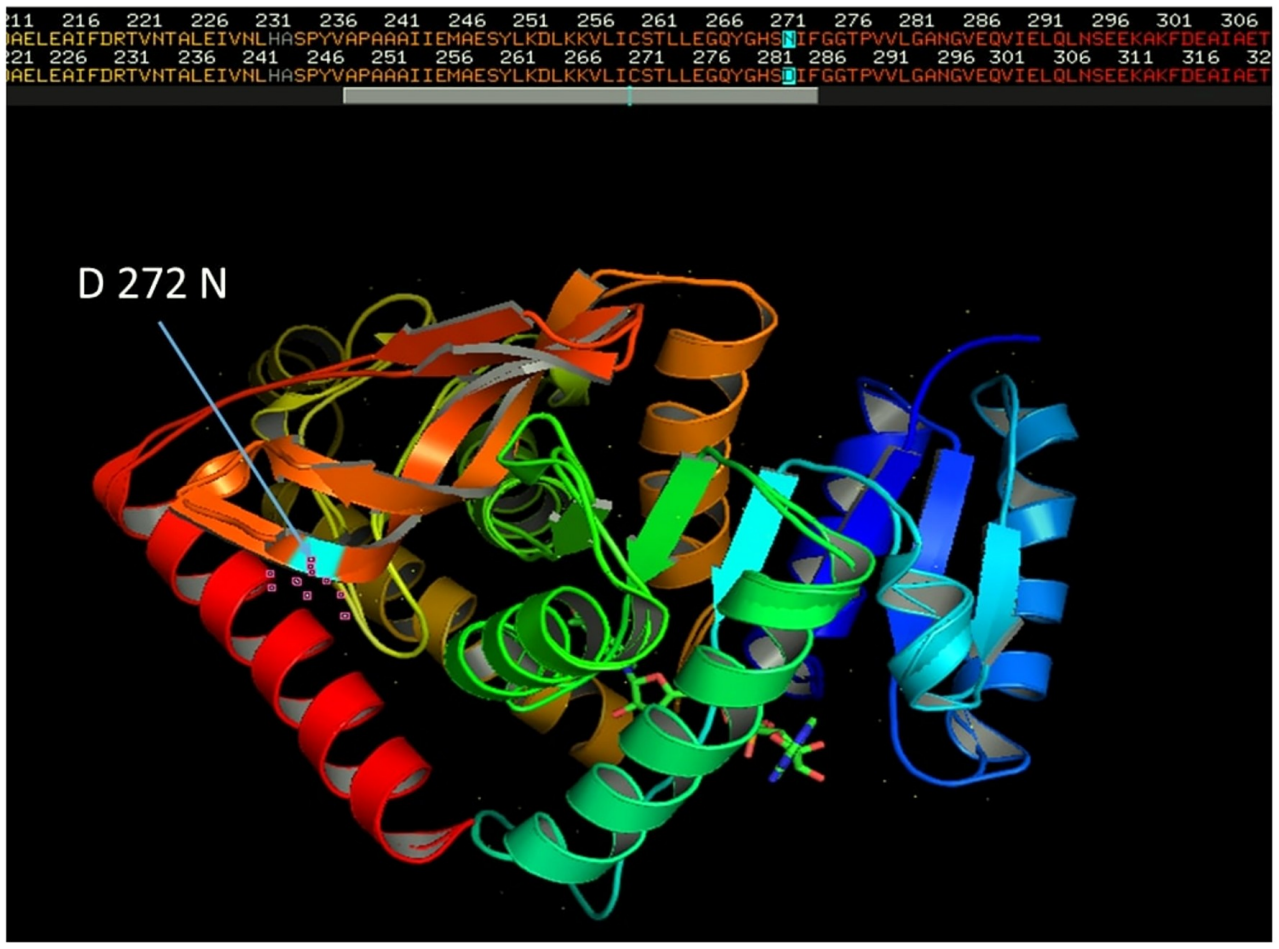
Structural alignment of predicted protein model of PfLDH with the reference crystal structure (PDB ID: 2A94) in PyMol. Highlighted region in protein showing reference structure fully superimposed on the predicted polymorphic PfLDH protein structure with RMSD value 0.056.

A total of 450 successfully sequenced good quality sequences were analyse to assess genetic variation in *Pfldh* gene. The amplified *Pfldh* gene of 951bp code for 316 amino acids fragment. After comparing the nucleotide sequence with reference sequence (PF3D7_1324900), four single nucleotide substitution were detected in five different samples at A 123 G, T 159 C, A 765 G and G 814 A position (Table 2). Non synonymous substitution at 814 position leads to amino acid change from D to N (Fig 3). Rest of the samples showed 100% nucleotide homology with the reference sequence. Predicted model of polymorphic PfLDH haplotype were generated by user template model in Swiss Model workspace. The QMEAN score of model is 0.59. Both the models matched significantly with crystal structure of LDH from *P*. *falciparum* (PDB Id: 2A94) with RMSD value 0.056 Å (Fig 4). No significant alteration in 3D structure of protein was observed.

### Sequence and structural analysis of *aldolase* genes from *P*. *falciparum* Indian isolates

**Fig 5:**
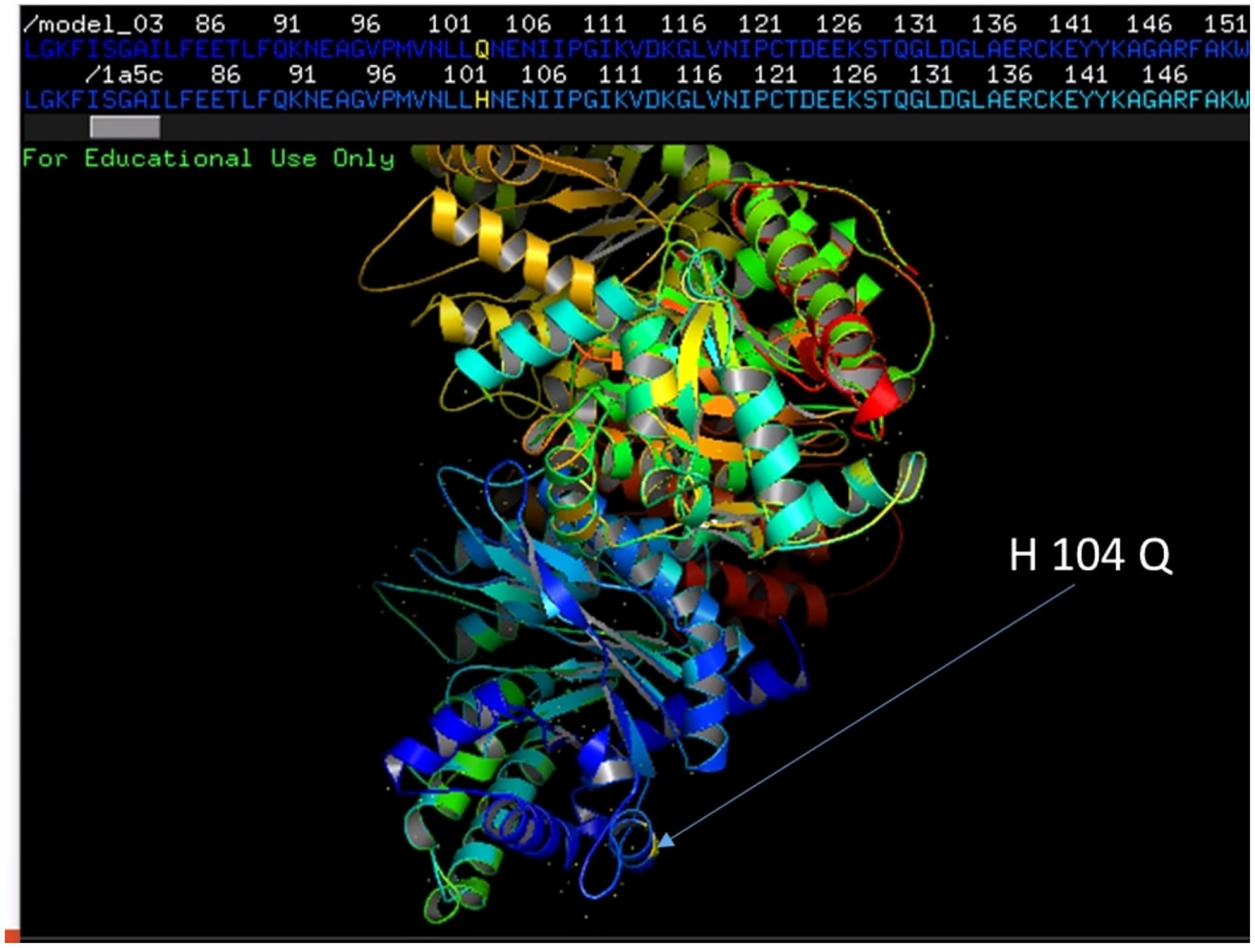
Structural alignment of predicted protein model of PfALDO with the reference crystal structure (PDB ID: 1A5C) in PyMol. Highlighted region in protein showing reference structure fully superimposed on the predicted protein structure of polymorphic haplotype with RMSD value almost 0.074.

A total of 464 samples were successfully sequenced and the sequences were aligned with the reference sequence (PF3D7_1444800) to analyse genetic variation in *Pf aldolase* gene. Eight single nucleotide substitutions were detected in eleven different positions C 156 T, C 234 T, C 312 G, A 444 T, A 630 G, T 729 A, C 753 T and A 819 C (Table 2). Only one non synonymous substitution at 312 position from C to G leads to amino acid change from H to Q (Fig 3). Rest of samples showed 100% nucleotide homology with the reference sequence. Predicted PfALDO model of polymorphic haplotype was generated in Swiss Model workspace using template with the E-value 0.0, Max Identity 99% and Query Coverage 100%. Model quality was estimated using QMEAN score which is -0.86. Both the models matched significantly with crystal structure of aldolase from *P*. *falciparum* (PDB Id: 1A5C) with RMSD value 0.074 Å (Fig 5).

### Purifying selection pressure on *pfgdh*, *pfldh* and *pfaldo* gene

The dN/dS ratio of *pfldh*, *pfaldo* and *pfgdh* haplotype showed that all three genes are under negative selection pressure (Table 3). No transversion substitution was observed in *pfldh* haplotype whereas transversion for *Pfgdh* haplotype was higher as compared to the transitions.

**Table 3:** Nucleotide diversity test of neutrality for *pfgdh*, *pfldh* and *pfaldo* gene.

Gene	No. of haplotype	dN	dS	dN/dS ratio	transition	transversion	S	θ	Π	Tajima’s D
**pfgdh**	**2**	**0.0**	**0.0044**	**0.0**	**0.0005**	**0.0005**	**2**	**0.000944**	**0.000944**	-
**pfldh**	**4**	**0.0005**	**0.0057**	**0.087719298**	**0.0017**	**0**	**4**	**0.002019**	**0.001682**	**-1.093799**
**pfaldo**	**8**	**0.0003**	**0.0061**	**0.049180328**	**0.0008**	**0.0008**	**8**	**0.002652**	**0.0016**	**-1.797517**

Abbreviations: dN = Average number of nonsynonymous mutation, dS = Average number of synonymous mutation S = Number of segregating sites, θ = hetrozygosity per site, π = nucleotide diversity, and D is the Tajima test statistic.

## Discussion

Diagnosis is critical for achieving the goal of malaria elimination set by the government of India by 2030. Rapid diagnostic tests (RDTs) are the major diagnostic tool particularly used in field and community surveys in malaria endemic countries with under-equipped health care infrastructures. The WHO has issued recommendations about the choice of RDTs, however selection of right RDT remains difficult for most users in endemic countries because of variation in targeted antigen in local parasitic population [34, 35]. One of the most important, but least studied of these factors is genetic variability of the target antigens of malaria parasite. [36, 37].

Most of the RDTs at present use pf*HRP2*, Plasmodial aldolase and Plasmodial lactate dehydrogenase as antigen for diagnosis of malaria parasite [38]. About 90% of RDTs targeting *Plasmodium falciparum* use HRP-2 as antigen. Though the exact function of HRP-2 and its homolog HRP-3 are not clearly known but the protein is water soluble, heat stable, expressed in parasitophorous vacuole or cytoplasm of parasite [18]. The *pfhrp-2* gene is located on chromosome 8 whereas *pfhrp-3* is located on chromosome 13. It is not an essential gene and parasite can survive even when the both the genes are partially or completely deleted. The reports of partial or complete deletion of pfhrp-2/3 across the malaria endemic regions in world are worrisome [39]. The *hrp-2* based RDTs fail to detect these parasites which continue to infect new hosts and cause malaria. The deletion *of pfhrp-2* and prevalence of malaria parasite lacking the gene necessitates the development of alternative RDTs/tools based on other antigens for malaria diagnosis. Keeping in mind, we selected the *P*. *falciparum* positive blood samples from the same sites where *hrp-2* deletion was detected [9] and the genetic diversity of *pfgdh*, *pfldh* and *Pfaldo* was analysed. Unlike the sequence polymorphism in *pfhrp*-2/3 reported by Bharti et al 2016 and 2017, *pfaldolase* and *pfldh* genes appear to be highly conserved. Aldolase is a glycolytic enzyme found in host tissues and malaria parasite. It shares 61–68% sequence similarity. The *P*. *falciparum* and *P*. *vivax* aldolase sequences are conserved and mostly employed for pan RDT as antigen. Likewise, out of eight nucleotide substitutions reported at different position of aldolase gene in this study only one substitution led to amino acid change (at codon 104). Despite having minor difference in the amino acid sequence, the structure of the protein did not change. However, Aldolase based RDTs are less popular because of low sensitivity due to less secretion of antigen [13,40].

In the absence of citric acid cycle, LDH is an important energy producing enzyme of glycolytic pathway of parasite. it is expressed in both sexual (gametocytes) as well as asexual stages of the parasite. Similar to aldolase, sequencing and comparative analysis of *pfldh* gene sequences showed 3 synonymous and 1 non synonymous (0.44%) mutations. Further, the amino acid sequence alignment of PfLDH and comparison of the protein structure with the reference in the PyMOL 1.7.4 shows that the 3D structure of PfLDH protein and region targeted in the RDT [40] remain conserved and the non-synonymous substitutions did not alter the enzyme structure. In contrast to our finding, Kelusker *et al*. reported no variability among sequences from a small number (n = 45) of Indian *P*. *falciparum* isolates collected from different study sites at Odisha, Karnataka and Goa [41]. However, the strength of our study is the analysis of at least 50–70 *P*. *falciparum* positive samples per site. A recent report about the analysis of genetic variation of *pfldh* gene from Thailand, India, Iran and Medagascar corroborates the conserved nature of *pfldh* gene [42]. However, *ldh* based RDTs are less sensitive. The poor sensitivity of LDH based RDTs is due to its inability in detecting low parasitemia and low sensitivity in high tropical temperature [43]. Further, recent sequence analysis from Thailand also supports our observation of conserved *pfldh* sequences.

Sequencing and analysis of *pfgdh* sequences shows that gene is highly conserved and only two synonymous SNPs were observed with no amino acid change among Indian isolates. Very little sequence diversity has been observed in *Pfgdh* in comparison to the low level of diversity in *ldh* and *aldolase* gene. Similarly, Seol B and his colleagues also reported that *gdh* gene of *P*. *vivax* from South Korea are highly conserved [44]. The neutrality analysis of pfgdh, pfldh and pfaldolase genes using tajima D test shows that all genes are under negative purifying selection and are conserved. The homohexameric PfGDH protein (Mw = 49.5 kDa, GDH1 PF3D7_1416500) shares only 23% overall amino acid sequence identity with the human counterpart [45] (HGDH, Uniprot gene- GLUD1) where as 69–86% identity with Pv GDH (Uniprot gene-PVX_085005, PVX_085625), 86% identity with PmGDH (Uniprot gene—PMALA_027050), 85% identity with PoGDH (Uniprot gene -POVCU1_039950) and 85% identity with PkGDH (Uniprot gene- PKH_133070) (S1 Table). The similarity of PfGDH with other plasmodium species may lead to cross reaction in areas where mixed malaria infection are found [23]. Though some studies have reported the PfGDH concentration in serum of malaria patients including in asymptomatic malaria, usually lies in nanomolar range [46], yet further studies on GDH expression in local clinical samples from malaria patients are required to test the efficacy of GDH as potential biomarker. The major limitation of the study is our inability in correlating the level of parasitemia with that of expression of *pfgdh*, *pfldh* and *pfaldolase* genes and their antigen levels in blood samples due to retrospective nature of the study. Our study provides scientific evidence for the conserved nature of *pfgdh* gene/protein sequences in Indian isolates which can be used as a potential biomarker for diagnosis of malaria. A recently published study has reported the development of DNA aptamer based biosensor targeting pfGDH and PLDH gene [45,47]. However, validation of the device needs to done in field conditions. In light of deletion of *pfhrp2/3* genes and poor sensitivity of *ldh* and *aldolase* based RDTs, it is imperative to explore the new diagnostic biomarker to supplement the arsenal of malaria diagnostics and to achieve the malaria elimination goal. This study creates basis for further evaluation of pfGDH based malaria diagnostics for future applications .in *pfhrp2/3* deleted areas.

## Supporting information

S1 Table Homology analysis of *P*. *falciparum* glutamate dehydrogenase. (DOCX)
