# Supplementary material for: Plasmodium falciparum glutamate dehydrogenase is genetically conserved across eight malaria endemic states of India: Exploring new avenues of malaria elimination
Source: PLoS One. 2019 Jun 14;14(6):e0218210. doi: 10.1371/journal.pone.0218210 (PMC6568416; doi:10.1371/journal.pone.0218210)
Supplement: S1 Table — (DOCX) [file pone.0218210.s001.docx]

***Plasmodium falciparum* Glutamate dehydrogenase is genetically conserved across eight malaria endemic states of India: exploring new avenues of malaria elimination**

Amreen Ahmad^1^, Anil Kumar Verma^1^,Sri Krishna^1^,Anjana Sharma^2^, Neeru Singh^1†,^ Praveen Kumar Bharti^1*^

^1^ ICMR-National Institute of Research in Tribal Health (NIRTH), Garha, Jabalpur, 482003, India

^2^ Department of P. G. Studies and Research in Biological Science, Rani Durgavati University, Pachpedi, Jabalpur 482001, Madhya Pradesh, India

^†^ Deceased on 19 August 2017

^*^ Corresponding author

E-mail: [saprapbs@yahoo.co.in](mailto:saprapbs@yahoo.co.in)

**S1 Table. Homology analysis of *P. falciparum* Glutamate dehydrogenase**

| **Sr. no** | **Species name** | **Accession no.** | **Query coverage** | **E value** | **identity** |
| --- | --- | --- | --- | --- | --- |
| 1 | *P.falciparum* 3D7 | XP_001348337.1 | 100% | 0.0 | 100% |
| 2 | *P. reichenowi* | XP_012765229.1 | 100% | 0.0 | 99% |
| 3 | *P. gaboni* | XP_018639242.1 | 100% | 0.0 | 96% |
| 4 | *P.vivax* Sal-I | XP_001616788.1 | 100% | 0.0 | 86% |
| 5 | *P. knowlesi* strain H | XP_002260827.1 | 100% | 0.0 | 86% |
| 6 | *P. malariae* | SBS89696.1 | 100% | 0.0 | 86% |
| 7 | *P. ovalewallikeri* | SBT45751.1 | 100% | 0.0 | 86% |
| 8 | *P. gonderi* | GAW83051.1 | 100% | 0.0 | 86% |
| 9 | *P. ovalecurtisi* | SBS87534.1 | 100% | 0.0 | 85% |
| 10 | *P. cynomolgi* strain B | XP_004224475.1 | 100% | 0.0 | 85% |
| 11 | *P. chabaudichabaudi* | SCM04229.1 | 95% | 0.0 | 63% |
